# Synthesis and characterization of surface-modified magnetic mesoporous silicate materials for phosphate adsorption

**DOI:** 10.55730/1300-0527.3638

**Published:** 2023-11-02

**Authors:** Ayşegül GENÇER, Burcu UYSAL KARATAŞ, Önder TOPEL, Nadir KİRAZ

**Affiliations:** 1 F.Yılmaz Sezer Vocational and Technical Anatolian High School, Antalya, Turkiye; 2 Department of Chemistry and Chemical Processing Technologies, Denizli Technical Sciences Vocational School, Pamukkale University, Denizli, Turkiye; 3 Department of Chemistry, Akdeniz University, Antalya, Turkiye

**Keywords:** Magnetic mesoporous material, modified MCM-41 absorbent, phosphate ion adsorption, absorption isotherm and kinetics

## Abstract

The magnetic mesoporous silica material, Mag-MCM-41, was synthesized by coating magnetite (Fe_3_O_4_) nanoparticles with a mesoporous material called MCM-41. Mag-MCM-41 and modified nanomaterials Mag-MCM-41-NN and Mag-MCM-41-NN-Fe^+3^ which were modified with aminopropyl functional groups. In water and wastewater, phosphate anions are considered significant contaminants due to their detrimental impact on the environment. They promote the growth of algae, leading to eutrophication. The purpose of this study is to investigate the removal of phosphate anions from aqueous solutions using modified magnetic silica particles. The Mag-MCM-41 material exhibits hexagonal properties and belongs to the class of “mesoporous” materials. It has a surface area of 923 m^2^.g^−1^, which was determined through N_2_ adsorption-desorption isotherms, FTIR, TEM, BET, and SAXS analysis. Kinetic and adsorption isotherm studies were conducted using Mag-MCM-41, Mag-MCM-41-NN, and Mag-MCM-41-NN-Fe^+3^ adsorbents to examine the behavior of phosphate anions. The kinetic and adsorption isotherm studies of phosphate anions revealed that the adsorption process on Mag-MCM-41, Mag-MCM-41-NN, and Mag-MCM-41-NN-Fe^+3^ adsorbents followed the chemical adsorption mechanism. Phosphate adsorption on all adsorbents occurred in a monolayer, and the MCM-41-NN-Fe^+3^ adsorbent exhibited the highest maximum adsorption capacity (qm) value of 112.87 mg.g^−1^ compared to the other adsorbents.

## 1. Introduction

Phosphorus is the eleventh most abundant element in the earth’s crust and is also essential for DNA, RNA and ATP in all living things. Phosphorus exists primarily in the geosphere as phosphate (PO_4_^3−^), which is the most prevalent form of phosphorus oxoanions. The term “orthophosphates” is commonly used to refer to phosphate. Almost every metallic element forms a bond with the orthophosphate anion [1,2]. Phosphate is used extremely and mixed with environmental waters as waste. It is very important to collect phosphates from the wastewater and discharge them before entering the environmental water since phosphorus is known as the main responsible for eutrophication in lakes. Eutrophication is a term used to describe a nutrient enrichment method, in which water changes from a nutrient-poor state (oligotrophic) to a nutrient-rich state (eutrophic). This situation leads to an accelerated growth of algae, which disrupts the balance of aquatic organisms and negatively impacts the water quality. The algae concentration and the trophic (nutritional) state of the lakes are closely related to the phosphorus level in the water [3]. The phosphorus concentration should be kept at a certain level to avoid eutrophication and to ensure the biological stability of the water. For example, the level of phosphorus concentration in streams, lakes and rivers was determined as <0.02 mg.L^−1^ by the U.S Environmental Protection Agency [4,5].

It is essential to control phosphate ion concentration in the water and many methods have been proposed for phosphate removal and recovery from water. Physicochemical precipitation and biological treatment methods are widely used for phosphate removal at the industrial level. As a result of advanced research, it has been suggested that the adsorption method is the more useful and economical method with advanced adsorbents that have high selectivity and removal capacity for phosphates [3,6,7].

The adsorbents should also be nontoxic, cost-effective, easily available, and recyclable. Common adsorbents are generally classified as activated carbon, nanomaterials, composites and nanocomposites, some other low-cost materials and materials of different sizes like alumina and silica. Silica is classified into two categories: nonporous and mesoporous. The main characteristics of mesoporous silica are controlled pore size, narrow distribution of pore size and high specific surface area [8–10]. Dinari and coworkers many times modified ordered mesoporous silica with different groups using different methods, and has been studied in many applications such as adsorbents for trace Cr^VI^ removal [11–13].

An adsorbent must have a high surface area or micropore volume and should also have a wide pore network where adsorbate molecules can enter. This shows that an advanced adsorbent should have a combination of two pores, a micropore, and a mesopore [14,15]. Mesoporous materials are particularly suitable as adsorbents due to their high specific surface areas, well-ordered pore radius distributions, and regular pore systems. These materials can be easily surface-modified based on their unique properties. As a result, mesoporous materials have found extensive applications as adsorbents for the removal of various organic and inorganic contaminants from water [4].

Scientific advances in the synthesis of mesoporous materials containing organic or inorganic nanoparticles represent an attractive field of nanoscience and nanotechnology [16]. With its adjustable pore diameters, well-ordered pore structure and high specific surface area, ordered mesoporous carbons are suitable for applications in many fields of modern science and technology [17]. Mohammadnezhad et al. was used modified mesoporous carbon for several process [17]. Recently, like mesoporous carbon, mesoporous silica materials have attracted attention in many fields such as adsorption, extraction, catalysis and drug delivery. Mesoporous silica materials have considerably been of interest in adsorption technology, especially the removal of organic and inorganic pollutants, dyes, and anions from aqueous solutions. In 2020, Dinari and coworkers studied on functionalized mesoporous fibrous silica nanospheres for dye adsorption and they got pretty good results [18].

The M41S family is a significant group of mesoporous materials, and it includes well-known members such as MCM-41, MCM-48, and MCM-50. MCM-41 (Mobil Crystalline Material) is a two-dimensional mesoporous material characterized by a hexagonal arrangement of mesopores in the p6mm space group. MCM-48, on the other hand, exhibits a three-dimensional pore structure formed by the cubic arrangement of mesopores in the la3d space group. MCM-50 differs from the other members as it consists of an unstable layered structure with a p2 space group. Among these materials, especially MCM-41 and MCM-48 have high thermal stability and adsorption capacity. MCM-41 is the most popular mesoporous material widely studied by researchers [19–21]. It is a silicate obtained by hydrothermal synthesis and liquid mold mechanism and it has a regular two-dimensional hexagonal channel-like pore arrangement and narrow pore size distribution. Pore diameters can be regulated in the range of 1.5–10 nm with varying alkyl chain lengths of the surfactant [22]. Pore volume is usually between 0.7–1.2 cm^3^.g^−1^. As a result of surface area measurements, it was determined that these materials have a surface area of approximately 1000 m^2^.g^−1^ [23–25].

Magnetic mesoporous materials are obtained by combining mesoporous silica materials with a magnetic core. These materials represent a novel class of functional materials with a high surface area, appropriate pore size, uniform pore size distribution, and can be easily separated from wastewater using magnets [26]. Wu et al. reported magnetic mesoporous materials that have attracted great attention in recent years, providing easy separation and recycling of the adsorbent after use [27]. In the literature studies, there are various studies on dye removal with magnetic MCM-41. Arica and coworkers were used modified and grafted magnetic MCM-41 composite particles for removal of direct dyes. In this study, two different textile dyes were removed from aqueous medium with high efficiency in a wide pH range of 4.0–7.0 [28]. Also, in the literature, it has been determined that there are studies on the use of magnetic mesoporous silica material in the removal of heavy metal ions, but there is no study on its use in the removal of phosphate ions. Zhang et al. used an amino-modified silica adsorbent containing Fe (III) ions at the surface to adsorb phosphate ions from an aqueous solution [29]. In the cited study the maximum adsorption capacity of the modified silica material was determined as 51.8 mg.g^−1^. Studies examined in the literature show that the adsorption capacity of mesoporous silica material is considerably increased when combined with magnetic iron oxide nanoparticles. This information has been determined through studies on the adsorption applications of heavy metal ions on magnetic mesoporous silica adsorbents [30].

The magnetic nanocomposite is an essential class of nanocomposites, with a magnetite core which is able to simply separated from the solution by using external magnate. Especially, magnetic nanocomposites with mesoporous silica have attracted increased attention recently. The novelty of present research stems from the coating of MCM-41 with Fe_3_O_4_ nanoparticles and also modified with aminopropyl functional groups and Fe^+3^ leading to novel magnetic nanocomposites for adsorption of phosphate anion. The other purpose of this study was to evaluate the effects of aminopropyl functional groups and Fe^+3^ on the modified MCM-41 particles. Adsorption kinetics and adsorption isotherm studies were carried out using Mag-MCM-41, Mag-MCM-41-NN, and Mag-MCM-41-NN-Fe^+3^ as adsorbents. In this study, the adsorption capacity, yield and adsorption rate of the adsorbents and also the effect of modification on adsorption were investigated. Additionally, several factors affecting the phosphate adsorption, such as different times or different concentrations, were also examined. The solution pH value was keeping in the range of 3.5–7.4. Because, in studies examining the effectiveness of modified MCM-41 in removing phosphate ions, it has been observed that keeping the solution pH value in the range of 3.5–7.4 increases the phosphate removal efficiency [31]. Comparative tests demonstrated that the Mag-MCM-41-NN-Fe^+3^ exhibited much superior phosphate adsorption capacity (112.9 mg.g^−1^) in all prepared samples.

We think that our study may contribute to the literature in terms of being the first findings showing that modified mag-MCM-41 can be effective in removing phosphate anion and that the synthesized substance can be used as an adsorbent. The efficiency of the synthesized adsorbents in the adsorption of different anions and dyes can be studied and may contribute to the literature in this sense.

## 2. Experimental

### 2.1. Materials

Iron (II) chloride tetrahydrate (FeCl_2_.4H_2_O; ≥99%, Sigma-Aldrich) and Iron (III) chloride hexahydrate (FeCl_3_.6H_2_O; 99%, Merck) were used in the synthesis of magnetite. Ammonium hydroxide solution (NH_4_OH; 28% Merck) was employed as an oxygen source in the synthesis of magnetite. Cetyl trimethyl ammonium bromide (CTAB, CH_3_(CH_2_)_15_N(CH_3_)_3_(Br); ≥98%, Sigma-Aldrich) was used to provide the hexagonal arrangement of the mesostructure in the synthesis of Mag-MCM-41. Sodium hydroxide (NaOH; 99%, Sigma-Aldrich) was used as a catalyst in the synthesis of mag-MCM-41. Tetraethylorthosilicate (TEOS, Si(OC_2_H_5_)_4_; 99%, Sigma-Aldrich) was used as a silica precursor compound in the synthesis of mag-MCM-41. 1-(2-aminoethyl)-3-aminopropyltrimethoxysilane (AEAPS, C_8_H_22_N_2_O_3_Si, 95% Abcr) was used for surface modification in the synthesis of mag-MCM-41-NN. Toluene (C_6_H_5_CH_3_; 99.8%, Sigma-Aldrich), Methanol (CH_3_OH; ≥99.8%, Sigma-Aldrich) and 2-propanol ((CH_3_)_2_CHOH; ≥99.8%, Sigma-Aldrich) were used as a solvent. Potassium dihydrogen phosphate (KH_2_PO_4_; ≥99%, Sigma-Aldrich) was utilized for the adsorption study of a phosphate anion. Antimony potassium tartrate (C_8_H_4_K_2_O_12_Sb_2_.xH_2_O; 99%, Merck), Ammonium molybdate tetrahydrate (NH_4_)_6_Mo_7_O_24_.4H_2_O; ≥99%, Acros), Ascorbic acid (C_6_H_8_O_6_; 99.7%, Merck) and sulfuric acid (H_2_SO_4_; 99.8%, Sigma-Aldrich) were used to prepare a reagent solution in adsorption studies of a phosphate anion.

### 2.2. Synthesis of magnetite (Fe_3_O_4_) nanoparticles

Fe_3_O_4_ nanoparticles were prepared by co-precipitation method according to the following reaction (Eq. 1) [30]:


(1) $${{\text{Fe}}^{+2}}_{\left(\text{aq}\right)}+2{{\text{Fe}}^{3+}}_{\left(\text{aq}\right)}+8{{\text{OH}}^{-}}_{\left(\text{aq}\right)}\to {\text{Fe}}_{3}{\text{O}}_{4\;(\text{k})}+4{\text{H}}_{2}{\text{O}}_{\left(\text{s}\right)}$$

Briefly, a mixture of 4.80 g of FeCl_3_.6H_2_O and 2.00 g of FeCl_2_.4H_2_O was dissolved in 30 mL of deionized water under vigorous stirring. The solution was then heated to 353 K under a nitrogen gas flow with vigorous stirring, and 20 mL of NH_4_OH was injected dropwise into the solution. Then, the solution was kept for stirring at 363 K under a nitrogen atmosphere for 2.5 h. At the end of the duration, the product was collected with a magnet and washed three times with water and methanol. Finally, the product was dried at 313 K for 24 h in a vacuum oven.

### 2.3. Synthesis of MCM-41 based magnetic mesoporous silica adsorbent

MCM-41-based magnetic mesoporous silica adsorbent was synthesized using methods as described previously [30,32]. The molar compositions used in the synthesis of magnetic mesoporous silica (Mag-MCM-41) material were 0.029, 0.313, 0.122, and 603.48 for SiO_2_, Fe_3_O_4_, NaOH, CTAB, and H_2_O, respectively. In the standard synthesis of magnetic mesoporous silica nanoparticles, 300 mg of Fe_3_O_4_ nanoparticles were sonicated in a mixture of 2 g CTAB, 7 mL of 2 M NaOH, and 480 mL of deionized water for 3 h. The mixture was heated at 353 K with stirring for 2 h and then, 10 mL of TEOS was added rapidly to the mixture. The final mixture was stirred for further 2 h. The reaction mixture was filtered, washed with deionized water and methanol, and dried at room temperature for 24 h. The dried product was then calcined at 873 K for 6 h at a heating rate of 1 K/min in the air to remove the surfactant. This synthesized material was referred to as Mag-MCM-41.

### 2.4. Synthesis of amino- and amino iron(III)-functionalized magnetic mesoporous silica adsorbents

The amino-functionalized and amino-iron-functionalized silicate nanomaterials, denoted as Mag-MCM-41-NN and Mag-MCM-41-NN-Fe^+3^, respectively, were prepared via the postsynthesis grafting method described by Anwander et al. and Zhang et al. [33,34]. Briefly, 1.00 g of calcined Mag-MCM-41 was stirred vigorously with 30 mL of dry toluene. Then, 5 mL (2 mmol) AEAPS was dropwise into the solution. The mixture was heated at 383 K under a nitrogen atmosphere for 6 h, and the functionalized material was subsequently filtered, washed with 2-propanol, and dried at 373 K. The brown powder was denoted as Mag-MCM-41-NN.

Mag-MCM-41-NN-Fe^+3^ was prepared by stirring 50 mM solution of FeCl_3_ in 2-propanol solution with 100 mg of Mag-MCM-41-NN for 2 h at room temperature. The obtained material was filtered, washed with 2-propanol, and dried at 383 K, resulting in the formation of a dark brown powder, which was designated as Mag-MCM-41-NN-Fe^+3^.

### 2.5. Phosphate ion adsorption experiments

In this study, the concentration of phosphate was determined using the ammonium molybdate spectrometric method, following the TSI (Turkish Standards Institution) standard (ISO 6878: 2004). In the standard method, a calibration curve was firstly created by plotting known phosphate concentrations against their corresponding spectrometric measurements. The phosphate adsorption values of the synthesized magnetic mesoporous silica adsorbents were then determined by measuring the remaining phosphate solution after adsorption experiments conducted at different times and concentrations.

In the preparation of the calibration curve with the TSE standard (ISO 6878: 2004) ammonium molybdate spectrometric method, firstly ascorbic acid and acid molybdate solutions were prepared as the reagent solution for the analysis of the remaining phosphate solution. Ascorbic acid solution (100 g.L^−1^) was prepared by dissolving 10g of ascorbic acid in 100 mL of deionized water. The acid molybdate solution was prepared through the combination of ammonium heptamolybdate tetrahydrate solution (130 g.L^−1^) and antimony potassium tartrate hemihydrate solution (3.5 g.L^−1^), and subsequently, the resulting solution was mixed with a 9 M H_2_SO_4_ solution. KH_2_PO_4_ solution containing 150 mg.L^−1^ PO_4_^−3^ was prepared as stock phosphate solution. KH_2_PO_4_ solution containing 6 mg.L^−1^ PO_4_^−3^(aq) was prepared from stock phosphate solution as standard phosphate solution. To prepare the calibration curve solutions, 1, 5, 9, 13, 17, 21, 25, 29, and 33 mL of KH_2_PO_4_ solution containing 6 mg.L^−1^ PO_4_^−3^(aq) were taken. Then, the solutions were approximately has been completed to 40 mL in a 50 mL flask and 1 mL of ascorbic acid solution and 2 mL of acid molybdate solutions were added to this solution. Finally, the solutions were completed to 50 mL and mixed for 10 min on a magnetic stirrer, then taken into the tub and their absorbances were measured with a Carry 100 brand UV-vis spectrophotometer in the range of 200–900 nm. For preparing the baseline solution, approximately 40 mL of ultrapure water was completed to 50 mL by adding 1 mL of ascorbic acid solution and 2 mL of acid molybdate solution.

Concentrations of phosphate solutions prepared in the range of 0.12–3.96 mg.L^−1^ for calibration graph. The calibration graph was obtained by plotting the concentrations of phosphate solutions against the absorbance values at the wavelength (880 nm) at which the maximum absorption occurred. In the linear regression analysis of the graph, it was determined that the molar absorptivity was 0.2296 mg^−1^.L.cm^−1^ and the regression coefficient was 0.9999 according to the Lambert-Beer equation. This shows that the calibration curve can be used with high accuracy and reliability to determine the maximum phosphate adsorption capacities of the samples [29].

In studies examining the efficiency of modified MCM-41 in removing phosphate ions, it was determined that keeping the solution pH value in the range of 3.5–7.4 increased the phosphate removal efficiency. Therefore, the pH range of 3.5–7.4 was studied in the whole study and experimental parts that depend on the change of pH parameter will be presented in future studies [31]. Experimental studies of adsorption isotherm and kinetic studies are given in the next sections.

### 2.6. Adsorption isotherm

Ten mg adsorbent was added into 10 mL phosphate solutions with various initial concentrations and suspension was shaken in the temperature-controlled shaker bath at 298K at 150 rpm for 24 h. Phosphate concentration ranges were determined as 0.1–30 mg.L^−1^ for mag-MCM-41, 150–500 mg.L^−1^ for mag-MCM-41-NN, and 25–1000 mg.L^−1^ for mag-MCM-41-NN-Fe^3+^. At the end of the adsorption process, the suspension was filtered with a 0.22 μm membrane syringe filter. The phosphate concentration of the filtered solution was analyzed using the standard method at 880 nm on a UV spectrophotometer.

The equilibrium adsorption capacities at equilibrium (qe) were calculated using Equation 2, and the adsorption isotherm data was fitted to the Langmuir (Eq 3), Freundlich (Eq. 4), and Temkin (Eq. 5) models [3].


(2) $$q_{e}=\frac{(c_{0}-c_{e})V}{m}$$

(3) $$\frac{c_{e}}{q_{e}}=\frac{1}{bq_{m}}+\frac{c_{e}}{q_{m}}$$

(4) $${lnq}_{e}={lnK}_{F}+\frac{1}{n}{lnC}_{e}$$

(5) $$q_{e}=K_{1}\;ln(K_{2})+K_{1}ln\;(C_{e})$$

where C_0_ (mg.L^−1^) is the initial phosphate concentration in solution, C_e_ (mg.L^−1^) is the phosphate concentration at equilibrium, q_e_ (mg.g^−1^) is the phosphate adsorption at equilibrium, q_m_ (mg.g^−1^) is the maximum adsorption capacity, b (L.mg^−1^) is the Langmuir constant, K_F_ (mg.g^−1^) is the Freundlich constant (related to Adsorption capacity), n is the Freundlich constant (related to Adsorption density), V (L)is the solution volume, m (g) is the adsorbent mass, K_1_ (L.mg^−1^) is the constant related to the heat of adsorption, K_2_ is the dimensionless Temkin isotherm constant.

### 2.7. Adsorption kinetics

Ten mg of adsorbent was added to a 10 mL solution containing 50 mg.L^−1^ of phosphate, and the suspension was shaken in a temperature-controlled shaker bath at 298 K and 150 rpm. The phosphate concentration was measured at time intervals (1, 5, 15, 30, 60, 120, 180, 240, 300, 360, 480 min). After the specified time, the suspension was filtered using a 0.22 μm membrane syringe filter, and the phosphate concentration in the filtered solution was analyzed at 880 nm using the standard method on a UV spectrophotometer.

The adsorption kinetic data was fitted using the Pseudo-first-order model (Eq. 6), Pseudo-second-order model (Eq. 7), and Elovich kinetic model (Eq. 8) [3]. By analyzing the curves generated by these equations, the experimental kinetic data of the samples were compared, and the most suitable kinetic model for the samples was determined.


(6) $$ln(q_{e}-q_{t})=ln(q_{e})-k_{1}\;t$$

(7) $$\frac{t}{q_{t}}=\frac{1}{k_{2}{q_{e}}^{2}}+\frac{1}{q_{e}}t$$

(8) $$q_{t}=\frac{1}{\beta }ln(\alpha \beta )+\frac{1}{\beta }ln(t)$$

where q_e_ and q_t_ (mg.g^−1^) are the adsorption capacity at equilibrium and time t, k_1_ (min^−1^) and k_2_ (g.mg^−1^.min^−1^) are the rate constants of pseudo-first-order and pseudo-second-order models, respectively, α (mg.g^−1^.min^−1^) is the initial adsorption rate, and β (g.mg^−1^) is the desorption constant.

### 2.8. Characterizations

The iron oxide (magnetite) nanoparticles were characterized using powder X-rays diffraction (XRD) (Bruker Axs D8 Advance model diffractometer with nickel-filtered CuKα radiation of wavelength 1.5406 A°, 2θ = 20° – 80°). Transmission electron microscopy (TEM) images of the iron oxide (magnetite) nanoparticles and magnetic mesoporous silica materials were obtained with a Zeiss Leo 906E brand TEM device. Measurements were carried out at 80 kV using the powder samples by dispersing them into ethyl ethanol with sonication and then a drop of the suspension was placed on a carbon-coated copper grid. FT-IR analysis was performed in the range of 400–4000 cm^−1^ in a Bruker Tensor brand FT-IR spectrometer with KBr pellet technique. Average pore diameter, pore volume and BET surface area of mesoporous material were determined by nitrogen (N_2_) adsorption technique with the Micromeritics MicroActive for TriStar II Plus 2.02 instrument. The Brunauer, Emmett and Teller (BET) method was used to determine the surface area while the average pore diameter and pore size distribution were determined by the Barrett-Joyner-Halenda (BJH) method. The hexagonal structure of the synthesized magnetic mesoporous silica materials was revealed using the SAXS technique. Analyzes were performed using the Kratky geometry Hecus System (Hecus X-ray systems, Graz, Austria) for small-angle X-ray scattering experiments. Copper Kα tube was used as an X-Ray tube and the wavelength of the tube used was λ = 1.54 A°. The energy of the beam falling on the sample is 2 kW (50 kV and 40 mA). Scattering patterns in the range of 0.004–0.55A° (scattering vector, q) were used as analysis results. The contents of C, H, and N in the synthesized mesoporous materials were determined by the elemental analysis technique. The analyses were carried out with a LECO CHNS-932 elemental analyzer. Two mg of sample was burned in the furnace at 1100 °C, and then element composition was measured using CHS-Infrared and N-Thermal conductivity detectors. To determine the concentration of phosphate, the ammonium molybdate spectrometric method was employed. The analysis was conducted using a Cary 100 model UV-Vis spectrophotometer, and the absorbance measurements were taken at a wavelength of 880 nm.

## 3. Results and discussion

### 3.1 Characterization of magnetic mesoporous silica adsorbents

**Figure 1:**
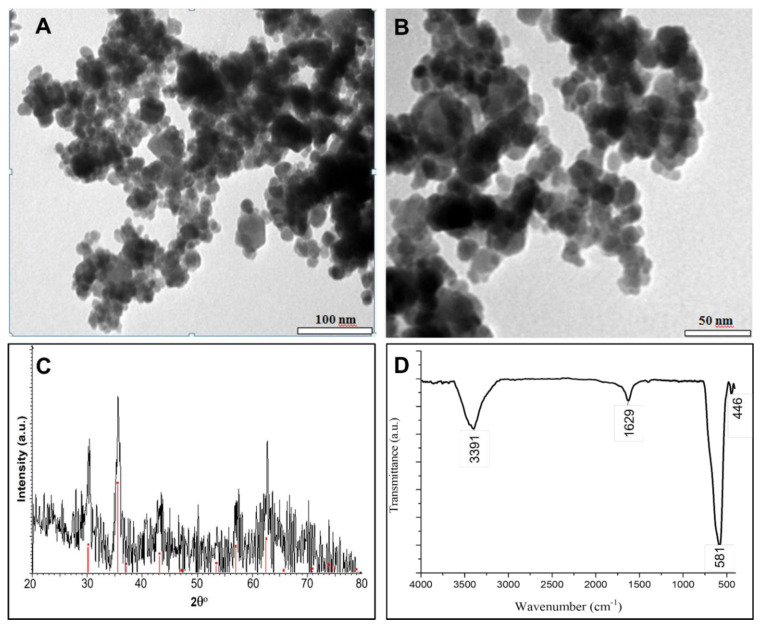
TEM images (A:100nm scale bar, B:50nm scale bar), XRD pattern (C), and FT-IR (D) measurement of Fe_3_O_4_ magnetic nanoparticles.

Magnetic mesoporous silica adsorbents were prepared by coating magnetic Fe_3_O_4_ nanoparticles with mesoporous silica (MCM-41). The objective of this synthesis was to enhance the adsorption efficiency of phosphate ions by utilizing the high surface area offered by mesoporous silica. For that, Fe_3_O_4_ nanoparticles were firstly synthesized, and their structure was characterized by TEM, XRD, and FTIR measurements as seen in Figure 1. The TEM image in Figure 1A and B reveals the presence of cubic Fe_3_O_4_ nanoparticles with an average size of 15 ± 0.49 nm. The crystal phase of the synthesized Fe_3_O_4_ particles was determined by XRD measurements. XRD diffraction pattern of the Fe_3_O_4_ particles in Figure 1C is compatible with the standard diffraction data belonging to the face-centered cubic crystal system structure with JCPDS Card No. 79-0419 shown as red dots in Figure 1C [32]. The peaks at 30.4°, 35.43°, 37.09°, 43.08°, 53.43°, 56.94°, and 62.57° were indexed to the lattice planes (220), (311), (222), (400), (422), (511), and (440) of the spinel structure, respectively. In a cubic 8-molecule unit cell of Fe_3_O_4_, eight Fe^+3^ cations occupy tetrahedral areas while every eight Fe^+2^ and Fe^+3^ cations host octahedral areas. This structure can be expressed as (Fe^3+^)_8_ (Fe^3+^Fe^2+^)_16_ (O_4_^2−^)_32_ [35,36]. The synthesized Fe_3_O_4_ particles were further analyzed using FTIR measurements, as shown in Figure 1D. The presence of the peak at 581 cm^−1^, corresponding to the Fe-O stretching vibrations, confirms the existence of Fe_3_O_4_ particles. [32]. Also, the O-H stretching peak at 3391 cm^−1^ and O-H bending peak at 1629 cm^−1^ correspond to the water molecules physically adsorbed in Fe_3_O_4_.

**Figure 2:**
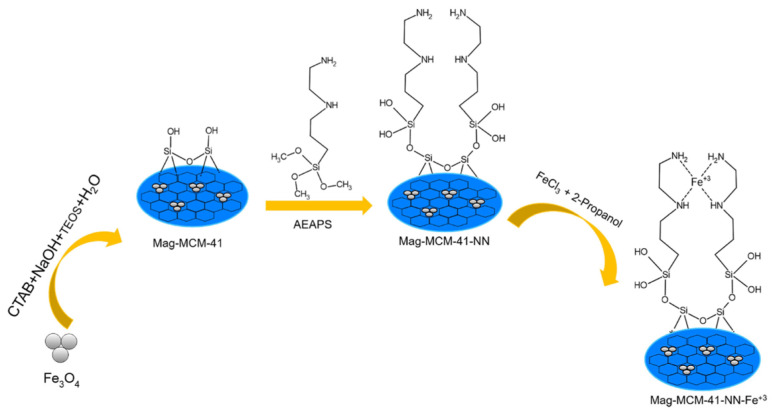
The synthesis route of the magnetic mesoporous materials.

As seen in Figure 2, three different mesoporous adsorbents were developed. The first adsorbent, named Mag-MCM-41, was obtained by coating the surface of the synthesized magnetic nanoparticle with mesoporous silica. The other two adsorbents, namely Mag-MCM-41-NN and Mag-MCM-41-NN-Fe^+3^, were prepared by modifying the surface of Mag-MCM-41 with AEAPS and introducing additional iron (III) complexing, respectively. The structure of the synthesized magnetic mesoporous particles was analyzed by FTIR, BET analysis, SAXS, TEM and elemental analysis measurements. FTIR measurements and elemental analysis confirmed the presence of silica in the mag-MCM-41 structure, while BET and SAXS analysis provided evidence for its mesoporous properties.

**Figure 3:**
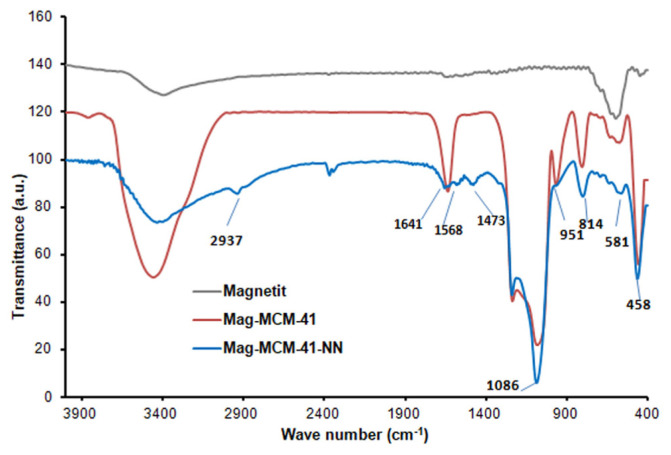
FTIR spectrum of Mag-MCM-41 and Mag-MCM-41-NN particles.

The FTIR spectra of Mag-MCM-41 and Mag-MCM-41-NN particles are given in Figure 3. Unlike the spectrum of Fe_3_O_4_ particles, the spectra of mag-MCM-41 and Mag-MCM-41-NN particles exhibit three characteristic peaks at 1086, 814, and 458 cm^−1^. These peaks correspond to the stretching vibrations of antisymmetric and symmetric Si-O-Si bonds, confirming the presence of the MCM-41 structure [37]. The modification of Mag-MCM-41 to form the mag-MCM-41-NN sample was confirmed by the presence of characteristic peaks in the FTIR spectrum. Specifically, the peak at 2937 cm^−1^ corresponding to the C-H stretching vibration and the peaks at 1568 cm-1 and 1473 cm^−1^ corresponding to the N-H asymmetric bending vibrations provide evidence for the successful introduction of amine groups, as shown in Figure 3. This shows the presence of Si-OH groups functionalized with alkyl amine groups in the structure after the modification of mag-MCM-41. At the same time, the shoulder peak of the Si-OH bond observed at 951 cm^−1^ indicates that −OH groups in the structure have been replaced by −NH_2_ groups [29].

Modification of Mag-MCM-41 to form Mag-MCM-41-NN particles was also analyzed by elemental analysis measurements. The elemental composition of Mag-MCM-41-NN particles was found as 14.560, 4.022, and 5.540% for C, H, and N %, respectively. It has been determined that the experimental value of % N obtained as a result of elemental analysis is compatible with the theoretical value of % N calculated as 5589 using the literature [34,38].

**Figure 4:**
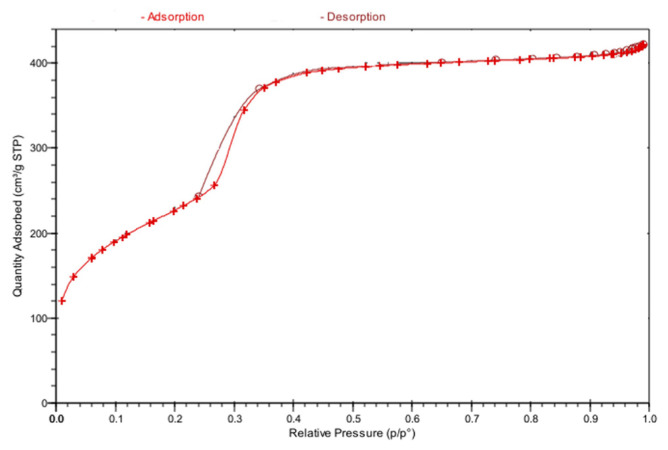
N_2_ adsorption-desorption isotherm of Mag-MCM-41.

**Figure 5:**
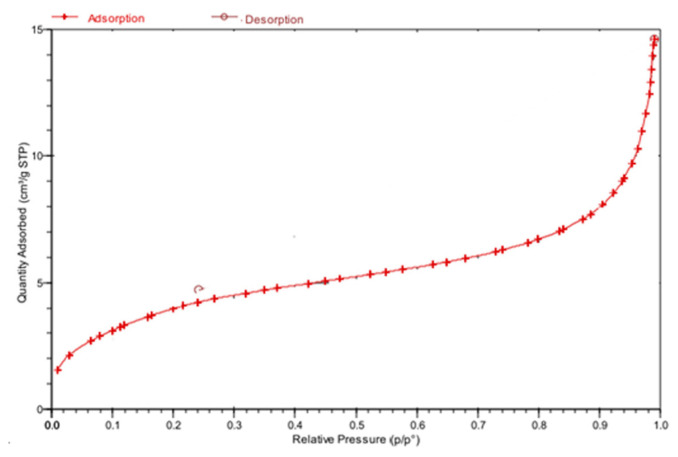
N_2_ adsorption-desorption isotherm of Mag-MCM-41-NN.

The N_2_ adsorption-desorption isotherms graphs obtained from BET analyses of the Mag-MCM-41 and Mag-MCM-41-NN particles are seen in Figure 4 and 5, respectively. The N_2_ adsorption-desorption isotherms of the calcined Mag-MCM-41 particles shown in Figure 4 are characteristic of a Type IV isotherm, according to the IUPAC classification. This result confirms that the synthesized Mag-MCM-41 belongs to the mesoporous material class [30]. In contrast, the N_2_ adsorption-desorption isotherm of the Mag-MCM-41-NN material presented in Figure 5 exhibits a Type III-like isotherm, indicating a weak interaction between the adsorbent and adsorbate [39].

The BET surface areas of the synthesized Mag-MCM-41 and Mag-MCM-41-NN materials were found to be 928.4 m^2^.g^−1^ and 15.06 m^2^.g^−1^, respectively. Additionally, the pore volumes of these materials were determined to be 0.700 cm^3^.g^−1^ for Mag-MCM-41 and 0.0148 cm^3^.g^−1^ for Mag-MCM-41-NN. The determined surface properties for Mag-MCM-41 are compatible with the literature data [30]. However, the surface properties of Mag-MCM-41-NN particles were determined significantly lower than those reported in the literature [29]. This observation suggests that the diamine groups may have occupied the pores, possibly filling the cores during the modification process.

**Figure 6:**
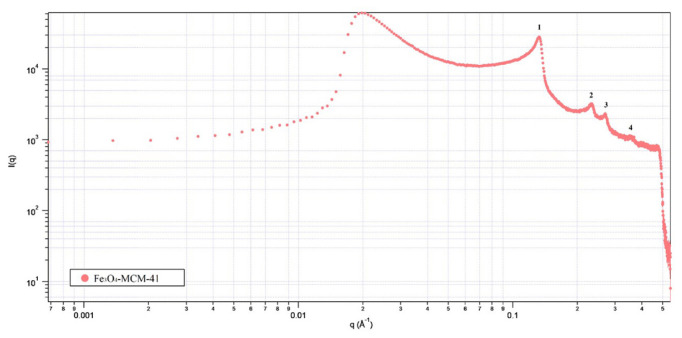
SAXS pattern of Mag-MCM-41.

The crystal structure of Mag-MCM-41 was further characterized by the small-angle X-ray scattering (SAXS) technique. The SAXS pattern is given in Figure. 6. The peaks in 2θ angles of 2.45°, 4.25°, 4.83°, and 6.44° denoted as the 1st, 2nd, 3rd, and 4th correspond to (100), (110), (200), and (210) hkl planes belonging the hexagonal structure (see Figure 6) [40]. Therefore, the mesoporous structure of synthesized Mag-MCM-41 was arranged hexagonally.

The lattice parameter (unit cell parameter), *α*_0_ and the wall thickness of the synthesized magnetic mesoporous silica adsorbent (Mag-MCM-41) were determined using SAXS data through Equations 9 and 10, respectively:


(9) α0=2d1003

(10) $$\int =\alpha_{0}-D$$

where *α*_0_ is the unit cell parameter, d_100_ is the interplanar distance, ∫ is the wall thickness, D is the pore diameter. The lattice parameter *α*_0_ of Mag-MCM-41 was determined as 5.55 nm by SAXS technique while the interplanetary distance (d_100_) was calculated as 4.81 nm using Eq. 9. The wall thickness of Mag-MCM-41 was calculated as 2.52 nm using Eq. 10. The determined parameters are all compatible with the literature data [37,41].

**Figure 7:**
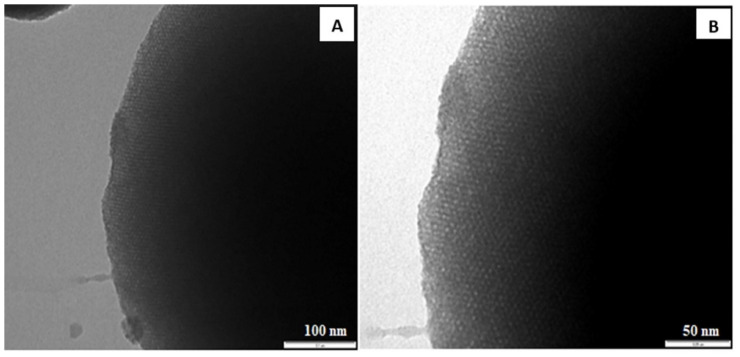
TEM images of Mag-MCM-41 (A:100nm, B:50nm).

The porous structure of Mag-MCM-41 was also characterized by TEM measurements. TEM images of Mag-MCM-41 seen in Figure 7 demonstrate the presence of regular hexagonal pores.

### 3.2. Phosphate ion adsorption studies on magnetic mesoporous silica adsorbents: Mag-MCM-41, Mag-MCM-41-NN, and Mag-MCM-41-NN-Fe^+3^

**Figure 8:**
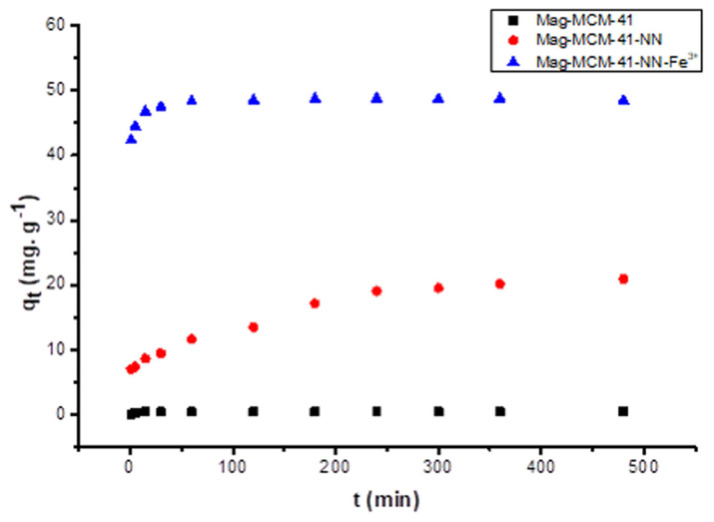
Time-dependent variation of phosphate adsorption capacities onto Mag-MCM-41, Mag-MCM-41-NN and Mag-MCM-41-NN-Fe^3+^ at 25 °C.

Time-dependent phosphate ion adsorption on the surface of the synthesized Mag-MCM-41 showed rapid equilibrium within 15 min, with a lower adsorption capacity of 0.42 mg.g^−1^. On the other hand, for Mag-MCM-41-NN, the equilibrium was established gradually over 480 min, with 85.7% of the maximum adsorption capacity reached in 240 min (see Figure 8). The adsorption capacity of phosphate ions in the equilibrium was determined as 20.9 mg.g^−1^. However, the adsorption equilibrium of phosphate ions on Mag-MCM-41-NN-Fe^3+^ was reached in 60 min with the highest adsorption capacity of 48.5 mg.g^−1^ (see Figure 8). The affinity of Fe^3+^ on the adsorbent surface to phosphate ions increased the adsorption capacity. The phosphate ion adsorption capacities of the synthesized Mag-MCM-41-NN and Mag-MCM-41-NN-Fe^3+^ were dramatically increased due to the modification from highly mesoporous Mag-MCM-41 adsorbent to the Mag-MCM-41-NN and Mag-MCM-41-NN-Fe^3+^ adsorbents with lower porosity and surface area interestingly. This shows that phosphate ion adsorption on the fabricated mesoporous adsorbents was mainly conducted by the affinity of the surface to phosphate ions rather than porosity and surface area. Hydrated phosphate ions are probably not able to penetrate the pores of the mesoporous material due to their larger volume. The affinity of the surfaces to phosphate ions increases in the order of the synthesized Mag-MCM-41, Mag-MCM-41-NN, and Mag-MCM-41-NN-Fe^3+^, which parallels the adsorption capacities in the equilibrium of 0.42, 20.9, and 48.5 mg.g^−1^, respectively.

**Figure 9:**
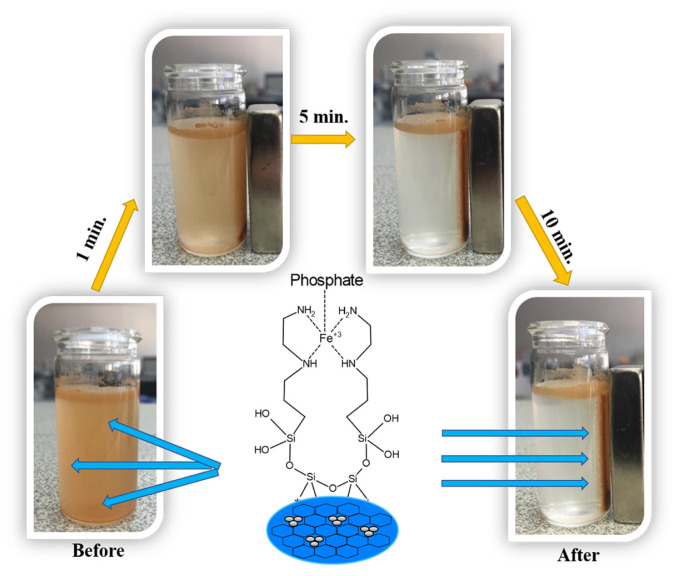
Separation of Mag-MCM-41-NN-Fe^3+^ from water by using a magnet in different min.

The sample (Mag-MCM-41-NN-Fe^3+^) was collected by the magnet in a short time (see Figure 9), indicating that the materials could be easily separated from the suspension system using magnetics.

**Figure 10:**
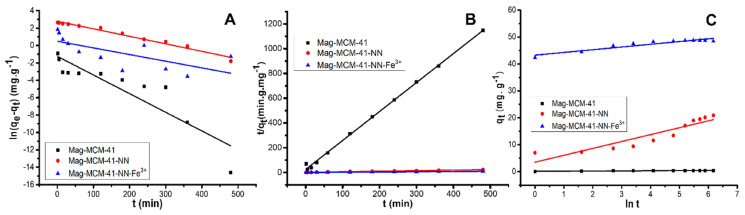
The fit of pseudo-first-order (A), pseudo-second-order (B) and Elovich (C) kinetic models for the adsorption of phosphate ion on Mag-MCM-41, Mag-MCM-41-NN, and Mag-MCM-41-NN-Fe^+3^ surfaces at 25 °C.

The kinetic data was evaluated by the pseudo-first-order (Eq. 6), pseudo-second-order (Eq. 7) and Elovich (Eq. 8) kinetic models to determine the kinetic mechanism for the adsorption of phosphate ions on the Mag-MCM-41, Mag-MCM-41-NN, and Mag-MCM-41-NN-Fe^+3^ adsorbents. The fitting of the kinetic models to the experimental data for the synthesized adsorbents is shown in Figure 10, and the corresponding kinetic rate constants and regression coefficient (r^2^) values are listed in Table 1. The regression coefficient (r^2^) values show that the most suitable kinetic model was the pseudo-second-order kinetic model for the adsorption kinetics of phosphate on Mag-MCM-41, Mag-MCM-41-NN, and Mag-MCM-41-NN-Fe^+3^ adsorbents. It indicates that the phosphate ion adsorption kinetics on the synthesized Mag-MCM-41, Mag-MCM-41-NN, and Mag-MCM-41-NN-Fe^+3^ adsorbents are mostly controlled by chemisorption processes, which supports the affinity-based adsorption mechanism discussed above. However, temperature-dependent supportive adsorption studies are needed to strongly emphasize the chemisorption process.

**Figure 11:**
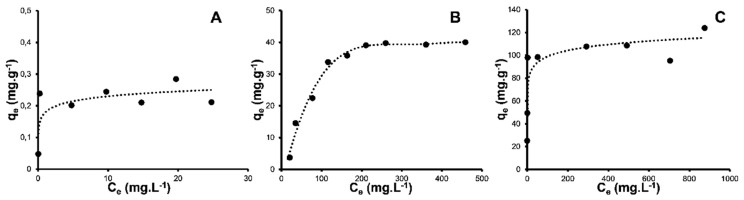
Langmuir isotherm plots for adsorption of phosphate anion on Mag-MCM-41 (A), Mag-MCM-41-NN (B), and Mag-MCM-41-NN-Fe^+3^ (C).

The isotherms studies for phosphate ion adsorption on the Mag-MCM-41, Mag-MCM-41-NN, and Mag-MCM-41-NN-Fe^+3^ adsorbents at 25 ^o^C are seen in Figure 11. Three common adsorption models (Langmuir, Freundlich, and Temkin isotherms) given by the Equations 3, 4, and 5, respectively, were applied to the experimental data, and the calculated parameters resulting from this analysis are presented in Table 2. Based on the r^2^ values in Table 2, it is clear that Langmuir isotherm is the most suitable model to explain the isotherm behavior for the phosphate ion adsorption on the Mag-MCM-41, Mag-MCM-41-NN and Mag-MCM-41-NN-Fe^+3^ adsorbents. This indicates that the phosphate ion adsorption on the magnetic mesoporous adsorbents occurs in a single layer, the surfaces of the adsorbents are homogeneous. Furthermore, all adsorption sites on the surface of the adsorbents exhibit equal adsorption energy.

The maximum adsorption capacities (q_m_) of Mag-MCM-41, Mag-MCM-41-NN, and Mag-MCM-41-NN-Fe^3+^ adsorbents were determined as 0.230, 43.94, and 112.9 mg.g^−1^, respectively. The maximum adsorption capacities (q_m_) of Mag-MCM-41-NN-Fe^+3^ are higher than the other adsorbents. This can be attributed to the modification of the mesoporous adsorbent with Fe^3+^ ions, which enhances the binding affinity of the adsorbent’s surface for phosphate ions and increases the electrostatic interaction between the surface and phosphate ions. The maximum adsorption capacity (q_m_) of Mag-MCM-41-NN adsorbent was also determined higher than that of Mag-MCM-41 since the surface of Mag-MCM-41-NN adsorbent is positively charged due to the protonation of the diamine groups on the surface at pH~5 in aqueous solution [4].

In summary, the Langmuir isotherm model best explains the adsorption behavior of phosphate ions on all three magnetic mesoporous adsorbents. The modification of adsorbents with Fe^3+^ ions and the presence of protonated diamine groups on the surface contribute to increased adsorption capacities for Mag-MCM-41-NN-Fe^+3^ and Mag-MCM-41-NN, respectively, compared to Mag-MCM-41.

### 3.3. Phosphate-adsorption mechanism

Phosphate adsorption mechanism has been studied extensively, and the findings can be summarized as follows: it was determined that phosphate ion adsorption on synthesized mesoporous adsorbents was primarily influenced by the affinity of the surface for phosphate ions rather than porosity and surface area. Adsorption kinetic studies also support the affinity-based adsorption mechanism. In isotherm studies, the fact that phosphate ion adsorption on mesoporous adsorbents occurs in a single layer can be interpreted as a factor that increases the affinity of the surface to phosphate ions since the surfaces are homogeneous. Modification of the mesoporous adsorbent with Fe^3+^ ions also increased the affinity of the surface for phosphate ions and the electrostatic interaction between the surface and phosphate ions [29].

### 3.4. Comparison of the phosphate removal capacity of MCM-41 and Mag-MCM-41 adsorbents with similar studies

The adsorption capacity values for phosphate ions from similar studies in the literature was summarized in Table 3.

In the literature, the highest phosphate ions adsorption capacity with MCM-41-CFA-10 adsorbent was attained as 64.2 mg.g^−1^ [42]. Additionally, it was observed that amine-modified materials show promising adsorption capacities, with the MCM-41-NN-La and MCM-41-NN-Fe^+3^ adsorbents demonstrating fairly good adsorption capacities of 54.3 mg.g^−1^ and 51.8 mg.g^−1^, respectively [29,33].

Upon examining similar studies in the literature alongside the current study, it was observed that the addition of Fe^+3^ modification to the amine-modified material led to a significant increase in adsorption capacity [29,44]. Notably, this study introduced magnetic properties to the MCM-41-NN-Fe^+3^ adsorbent for the first time, and its phosphate ions adsorption capacity was investigated. Comparing the results from the literature and this study, the synthesized adsorbent (Mag-MCM-41-NN-Fe^+3^) exhibited an exceptionally high adsorption capacity for phosphate ions when magnetic properties were incorporated.

In fact, the Mag-MCM-41-NN-Fe^+3^ adsorbent achieved the highest adsorption capacity value of 112.9 mg.g^−1^, surpassing all other similar materials investigated (as shown in Table 3). This significant enhancement in adsorption capacity highlights the potential and effectiveness of the magnetic mesoporous adsorbent in capturing phosphate ions, making it a promising candidate for phosphate removal applications.

## 5. Conclusions

In this study, magnetic ordered mesoporous silica materials, namely Mag-MCM-41, Mag-MCM-41-NN, and Mag-MCM-41-NN-Fe^3+^, were successfully synthesized. Comprehensive surface and structure-property characterizations were performed using FTIR, TEM, XRD, BET, elemental analysis, and SAXS analysis. The kinetic and adsorption studies of the phosphate ion on the fabricated Mag-MCM-41-NN and Mag-MCM-41-NN-Fe^3+^ adsorbents were carried out using the synthesized adsorbents. The results confirmed that the spherical MCM-41 particles were successfully modified with aminopropyl functional groups and Fe^+3^. Moreover, the surface area and average pore diameters of the particles were affected by the inherent properties of modified functional groups such as their side chains. In addition, modification with functional groups causes the reduction of surface areas and pore diameters, which is also evidence of surface modification. These magnetic-ordered mesoporous silica adsorbent materials proved to be effective for the removal of phosphate anions from aquatic solutions in the pH range 3.5–7.4.

The presence of Fe^+3^ ions on the adsorbent accelerated the adsorption process by creating a more active bonding medium with the phosphate ions. The pseudo-second-order kinetic model was found to be the most suitable model, indicating that the adsorption in this study follows a chemical adsorption mechanism. Moreover, the experimental isotherm data for phosphate adsorption fit well with the Langmuir isotherm model. The experimental isotherm data of phosphate adsorption was more suitable for the Langmuir isotherm model. When the Langmuir isotherm parameters are examined, the maximum adsorption capacity (q_m_) value was found to be the highest (112.9 mg.g^−1^) in Mag-MCM-41-NN-Fe^+3^. This showed that the modification of the material with Fe^+3^ ion increased the adsorption of the phosphate ion. In conclusion, compared with the literature results, Mag-MCM-41-NN-Fe^+3^ adsorbent has the highest adsorption capacity for the adsorption of phosphate ions with its maximum adsorption capacity value of 112.9 mg.g^−1^.

## Figures and Tables

**Table 1:** Kinetic parameters for the adsorption of phosphate ion onto Mag-MCM-41, Mag-MCM-41-NN and Mag-MCM-41-NN-Fe^+3^ adsorbents at 25 °C

*Pseudo-first-order kinetics*			

Adsorbents	k_1_ (10^−3^min^−1^)		R^2^
Mag-MCM-41	21.4		0.7823
Mag-MCM-41-NN	8.64		0.9747
Mag-MCM-41-NN-Fe^+3^	6.87		0.4239

*Pseudo-second-order kinetics*			

Adsorbents	k_2_ (10^−3^ g.mg^−1^.min^−1^)	h (mg.g^−1^.min^−1^)	R^2^

Mag-MCM-41	215.99	0.03949	0.9978
Mag-MCM-41-NN	1.4137	0.6643	0.9831
Mag-MCM-41-NN-Fe^+3^	99.229	234.74	0.9999

*Elovich kinetics*			

Adsorbents	α (mg.g^−1^.min^−1^)	β (g.mg^−1^)	R^2^

Mag-MCM-41	0.4449	18.149	0.7652
Mag-MCM-41-NN	10.092	0.3900	0.8379
Mag-MCM-41-NN-Fe^+3^	0.9994	1.0234	0.8744

**Table 2:** Langmuir, Freundlich and Temkin isotherm parameters for phosphate adsorption on Mag-MCM-41, Mag-MCM-41-NN and Mag-MCM-41-NN-Fe^+3^ adsorbents

*Langmuir isotherm*			

Adsorbents	q_m_(mg.g^−1^)	K_L_ (L.mg^−1^)	R^2^
Mag-MCM-41	0.230	174.5	0.9389
Mag-MCM-41-NN	43.94	0.030	0.9968
Mag-MCM-41-NN-Fe^+3^	112.9	0.064	0.9564

*Freundlich isotherm*			

Adsorbents	1/n	K_F_ (mg.g^−1^)	R^2^

Mag-MCM-41	0.1916	0.1827	0.2965
Mag-MCM-41-NN	0.9038	0.2833	0.8419
Mag-MCM-41-NN-Fe^+3^	0.1253	56.47	0.7166

*Temkin isotherm*			

Adsorbents	K_1_ (L.mg^−1^)	K_2_	R^2^

Mag-MCM-41	0.0388	451.4	0.1153
Mag-MCM-41-NN	11.75	0.1007	0.9474
Mag-MCM-41-NN-Fe^+3^	7.858	6151	0.6258

**Table 3:** Comparison of phosphate removal capacity for MCM-41 and Mag-MCM-41 adsorbents with different modifications.

Adsorbents	Adsorption capacity (q_m_) (mg.g^−1^)	Reference
MCM-41-NH_2_	45.2	[15]
MCM-41-NN-Fe^+3^	51.8	[29]
MCM-41- ammonium functional	20	[31]
MCM-41-NN-La	54.3	[33]
MCM-41-CFA-10	64.2	[42]
La25M41	20	[43]
MCM-41-Fe–EDA-SAMMS	43.4	[44]
MCM-41-rice husk composite	21	[45]
MCM-41-ammonium functional	20	[46]
Mag-MCM-41	0.230	This study
Mag-MCM-41-NN	43.90	This study
Mag-MCM-41-NN-Fe^+3^	112.9	This study
